# Transvaginal Photobiomodulation for the Treatment of Chronic Pelvic Pain: A Pilot Study

**DOI:** 10.1089/whr.2021.0097

**Published:** 2021-11-23

**Authors:** Ralph Zipper, Brian Pryor, Georgine Lamvu

**Affiliations:** ^1^ Zipper Urogynecology Associates and Laser Gyn Institute, Melbourne, Florida, USA.; ^2^ DJO Global, New Castle, Delaware, USA.; ^3^ University of Central Florida College of Medicine, Orlando, Florida, USA.

**Keywords:** chronic pelvic pain, photobiomodulation, low-level laser therapy

## Abstract

***Background:*** Chronic pelvic pain (CPP) is a common and debilitating condition that affects millions of U.S. women. Most treatments are ineffective and innovative new therapies are desperately needed. Large, controlled studies show that photobiomodulation (PBM) can reduce pain in patients with other chronic pain conditions, such as low back pain, neck pain, and fibromyalgia. The objective of this pilot study was to determine if transvaginal PBM (TV-PBM) can reduce pain in women with CPP.

***Methods:*** We conducted a before and after, observational, pilot study. Patients completed the Short Form-McGill Pain Questionnaire (SF-MPQ) at baseline, 1 week, 3 months, and 6 months after nine treatments of TV-PBM. Clinicians completed the Clinical Global Impression Scale (CGI) assessing patient illness severity at the same time. Wilcoxon rank-sum *t*-tests and effect size using Cohen's *d* coefficient (low effect size if *d* < 0.2, medium if 0.2 < d > 8, and high if *d* > 0.8) was used to measure degree of pain improvement, which was also considered clinically significant if pain reduction was >30%.

***Results:*** Thirteen women completed 9 treatments, and 10 women were successfully followed to 6 months. At baseline, the mean SF-MPQ score was 19.7 (standard deviation [SD] ± 5.9). Compared with baseline, 60% improved; the mean SF-MPQ score decreased to 10.0 (SD ±7.5, *p* = 0.004, *d* = 1.6) at 1 week after treatment, to 9.7 (SD ±7.9, *p* = 0.005, *d* = 1.7) at 3 months, and 8.2 (SD ±8.1, *p* = 0.002, *d* = 1.9) at 6 months.

***Conclusion:*** Transvaginal PBM provided significant and sustained pain relief to women with CPP up to 6 months. Further controlled studies are needed to confirm these findings, however, in this initial pilot, TV-PBM shows promise.

## Introduction

Chronic pelvic pain (CPP) affects as many as 15% of U.S. women, yet less than 5% have access to a pelvic pain specialist.^1–3^ CPP is defined as pain perceived to originate in the pelvis, typically lasting longer than 6 months that is associated with significant negative cognitive, behavioral, sexual, and emotional consequences, as well as symptoms suggestive of lower urinary tract, sexual, bowel, myofascial, or reproductive organ dysfunction.^4^ Persons with CPP usually present with severe pain that interferes with daily activities and results in the frequent use of medical resources.^5,6^ The U.S. economic costs of CPP were estimated in 1996 at $2.8 billion annually, but more recent estimates that include costs of all conditions associated with CPP, indicate that costs exceed $289 billion.^1,7,8^

Identifying the cause of pain is challenging because CPP can be associated with various conditions, including interstitial cystitis/bladder pain syndrome (IC/BPS), irritable bowel syndrome (IBS), endometriosis, and vulvodynia.^9^ In settings where women with CPP are routinely evaluated with a standardized pelvic examination, 50%–90% also have pain from pelvic musculoskeletal structures.^10–12^ Research shows that multiple pain syndromes, such as endometriosis, IBS, IC/BPS, and myalgias, often coexist in the same patient and 40%–70% of women with CPP have more than one cause for their pain.^8,9,13–15^

Often, pelvic pain specialists must choose from multiple treatment options, typically used in combination that may have limited effectiveness and multiple side effects. Although the Food and Drug Administration (FDA) has approved condition-specific pharmacotherapies for IBS, BPS, and endometriosis, those therapies come with substantial costs and side effects while minimally improving chronic pain. Surgical interventions for CPP, such as conservative resection of endometriosis, neurolysis, and hysterectomy, are invasive, costly, and have high rates of pain recurrence, especially in patients with overlapping pain and mental health comorbidities.^16–18^

Nearly 80% of patients with CPP also have signs of pelvic floor dysfunction and myalgia, therefore, treatment paradigms recommend that pelvic physical therapy (PT) be combined with other management modalities resulting in complex multimodal therapeutic plans. However, a 2019 systematic review of PT interventions for CPP concluded that, due to heterogeneity and poor methodologic quality, the evidence does not yet support the use of PT for CPP.^19^ Lack of standardized treatment protocols, poor compliance with therapy, and lack of access to specialized therapists, are barriers to using pelvic PT for treatment of CPP.

The most recent summary review of data, a 2012 report from the Agency of Healthcare Research and Quality entitled ‘noncyclic CPP therapies for women: comparative effectiveness,’ could not identify any effective treatments for CPP.^20^ Nearly a decade later, there is still no single therapy that can address the multiple components of CPP. Innovative therapies are desperately needed.

Photobiomodulation (PBM), previously referred to as low-level laser therapy (LLLT), is a form of near-infrared (NIR) light therapy. The energy emitted by LLLT devices is absorbed by chromophores, molecules in tissue capable of absorbing particular wavelengths of light. Examples of human chromophores include melanin, red blood cell hemoglobin, water, and the mitochondrial chromophore cytochrome c oxidase (COX). Mitochondria are considered “power plants” within cells because they can use oxidative phosphorylation to convert food and oxygen into energy in the form of adenosine triphosphate (ATP).^21^

Although the processes by which PBM affects tissue and inflammation are not yet fully understood, scientists have shown that PBM dissociates nitric oxide from COX in the mitochondrial membrane, resulting in relaxation of smooth and skeletal muscle and improved circulation to oxygen-deprived tissues.^21^ Mitochondrial activation can also enhance cell proliferation (of fibroblasts, keratinocytes, endothelial cells, and lymphocytes), neovascularization, promote angiogenesis, and collagen synthesis to help in acute and chronic wound healing. At certain levels (dosing) PBM can lead to nociceptor blockage and pain relief as well as reduction of local edema and inflammation.^21^ Randomized clinical trials and systematic reviews confirm that PBM can safely improve pain and reduce inflammation in musculoskeletal chronic conditions such as low back pain, fibromyalgia, as well as knee and shoulder pain.^22–24^

Given its effect on myofascial and visceral tissue, we speculated that transvaginal PBM (TV-PBM) may benefit patients with CPP, where inflammation and hypertonic muscular dysfunction contributes to pain. The primary endpoint of this study was to assess the effect of TV-PBM on pain severity in women with CPP. The secondary endpoint of this study was to assess loss of effect over time. Since TV-PBM is a novel therapy, we also wanted to assess compliance and safety.

## Methods

We conducted a 6-month, prospective, single-center, before and after, observational pilot study. The protocol was IRB approved by the New England IRB (NE-IRB 09-152) on August 19, 2009. The study was conducted at Zipper Urogynecology Associates, a free-standing urogynecology center in central Florida. Women were enrolled in the study only if they were 21 years or older, and fulfilled American College of Obstetricians and Gynecologists' (ACOG) criteria for CPP. To be included in the study, patients had pain longer than 6 months, had failed previous treatments, and were referred to the urogynecology center for management of CPP. Pregnant patients, those on light sensitizing drugs, and those with pelvic neoplasia were excluded.

**FIG. 1.**
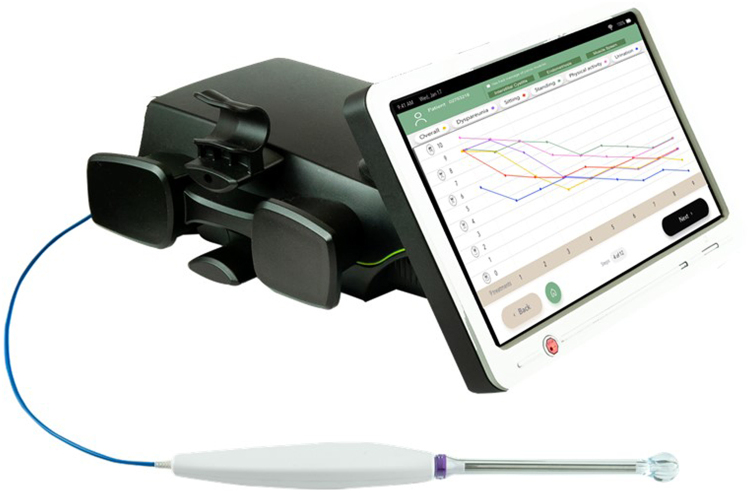
Photobiomodulation device with transvaginal probe.

After completing the informed consent, patients completed medical history and pain questionnaires, followed by gynecological examination, pregnancy test, and urinalysis. Following initial gynecological evaluation and confirmation of negative diagnostic tests, patients were treated with the SoLá Pelvic Therapy (Uroshape, LLC, Fig. 1) transvaginal PBM system (TV-PBMS). Dosing was optimized based on the available LLLT peer-reviewed medical literature on therapeutic irradiance, the surface irradiance created by the TV-PBMS, and the estimated mean vaginal surface area.^25–32^ Patients were treated at power settings of 5–8 W for a total of 3000 to 3500 Joules. For each treatment, the sterile, biocompatible NIR translucent SoLá TV-PBM probe (Fig. 1) was gently inserted to the apex of the vagina, and the laser was activated. The probe was then moved slowly from apex to introitus for the treatment duration, after which the laser was deactivated and the probe removed. Treatments were repeated on the following 2 days and then semiweekly until nine treatments were completed as approved by the NE-IRB. Patients were instructed to report any adverse events immediately.

Sample size was calculated for the primary outcome of interest, reduction in overall pelvic pain. Since this was a pilot study, and there is no previously published data to guide us, sample size could not be precisely calculated, however, we estimated sample size based on the assumption that an average of 20% improvement in raw clinical scores could be detected in as few as 20 patients (alpha 0.05, power 80%).

Patients completed a Short Form-McGill Pain Questionnaire (SF-MPQ)^33^ at baseline, and at 1 week, 3 months, and 6 months after the final treatment. The SF-MPQ ranks severity of pain symptoms on a numeric scale with 0 = none, 1 = mild, 2 = moderate, and 3 = severe.

The 15 pain descriptors that can be rated are throbbing, shooting, stabbing, sharp, cramping, gnawing, hot-burning, aching, heavy, tender, splitting, tiring/exhausting, punishing, sickening, and fearful. The pain scores were calculated from the sum of the intensity rank values for each of the words used to describe the pain.^33^ For example, if a patient selected 3 for aching, 2 for fearful, 3 for tender, and 0 (or did not rank) for all other descriptors, then their summative pain score is calculated as 8 out of 45 maximum points. The more pain descriptors that are ranked, the higher the pain score. If a patient was missing data for a time point, the highest pain score recorded was assumed to be their final pain score. For example, if a patient had a pain score of 19 at 6 months, but they had missing data at 3 months, then 19 was assumed to be their final pain score and this was imputed into the 3-month data field. If a patient had a score of 19 at 3 months, but their 6-month pain value was missing, then 19 was carried forward and imputed into their 6-month pain score.

For each patient at each visit, clinicians completed a Clinical Global Impression (CGI)^34–36^ assessment of the patient. This scale allows clinicians to rank the patient's illness severity on a 7-point Likert scale, where 1 = normal, not ill, 2 = borderline ill, 3 = mildly ill, 4 = moderately ill, 5 = markedly ill, 6 = severely ill, and 7 = among the most extremely ill patients. The CGI also includes a global improvement scale that ranks improvement as 0 = very much improved, 2 = much improved, 3 = minimally improved, 4 = no change, 5 = minimally worse, 6 = much worse, and 7 = much worse.

Lastly, the CGI includes a measure of therapeutic effect that considers both symptoms and side effects and is scored as 1 = unchanged or worse, 2 = minimal, 3 = moderate (decided improvement or partial remission of symptoms), and 4 = marked (nearly complete resolution of symptoms). Any therapy that in any way interferes with a patient's functioning reduces the therapeutic effect core.

Means and frequencies (%) were used for the initial descriptive analysis, and Wilcoxon signed-rank test was used to compare pain levels (mean SF-MPQ scores) and CGI scores for each category at baseline and compared with pain levels and scores at 1 week, 3 months, and 6 months. Statistical significance was set at a *p*-value <0.05. We tested the hypothesis that compared with baseline, there would be (1) a decrease in mean pain severity and illness severity, and (2) an increase in efficacy score. Clinically significant improvement was defined as greater than 30% in the mean pain score.^35,36^ Effect size, an additional measure used to describe degree of improvement, was measured using a single sample Cohen's *d* coefficient and interpreted as low effect size if *d* < 0.2, medium if 0.2 < *d* < 0.8, and high if *d* > 0.8.

## Results

Sixteen women were screened to participate in the study; 14 underwent the first treatment and 13 completed all 9 treatments. The mean age of the women was 62.8 years (standard deviation [SD] = 8.4, 47–75). No adverse events were reported or identified. One patient was missing data at the 1-week evaluation, one patient had missing data at the 3-month evaluation, and one patient had missing data at the 3- and 6-month evaluation.

### Clinical global impression

**FIG. 2.**
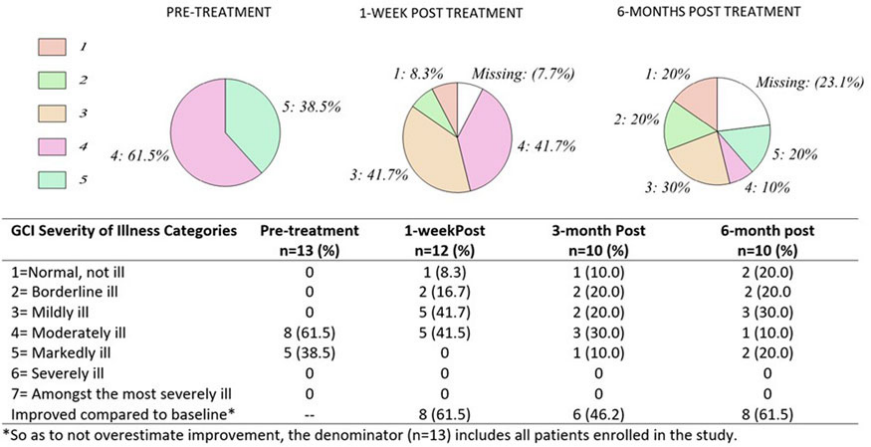
Percent of patients in each severity of illness category on the Clinical Global Impression (CGI) scale before and after treatment. CGI, Clinical Global Impression scale.

**FIG. 3.**
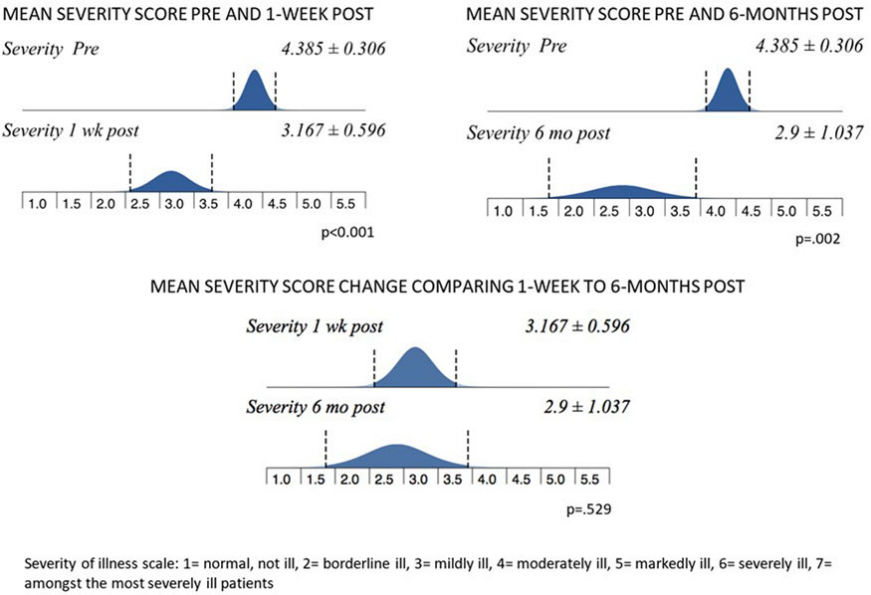
Mean severity of illness scores on the Clinical Global Impression scale before and after treatment.

The percentage of patients and corresponding severity of illness is depicted in Figure 2 for all time points. Mean severity scores from baseline compared with 1 week and 6 months were significantly decreased and shown in Figure 3. There was no significant difference between 1 week post-treatment and 6 months post-treatment (no loss of effect). For assessment of global improvement, a score of 4 (no change/improvement) was considered baseline.

**FIG. 4.**
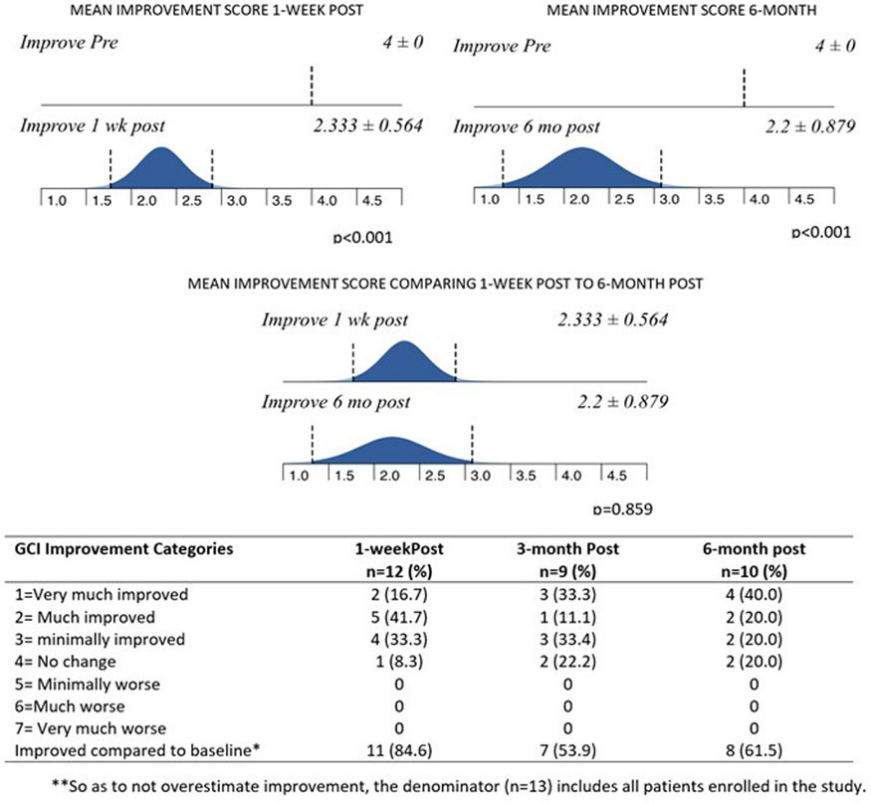
Changes in global improvement scores from pre- to post-treatment.

**FIG. 5.**
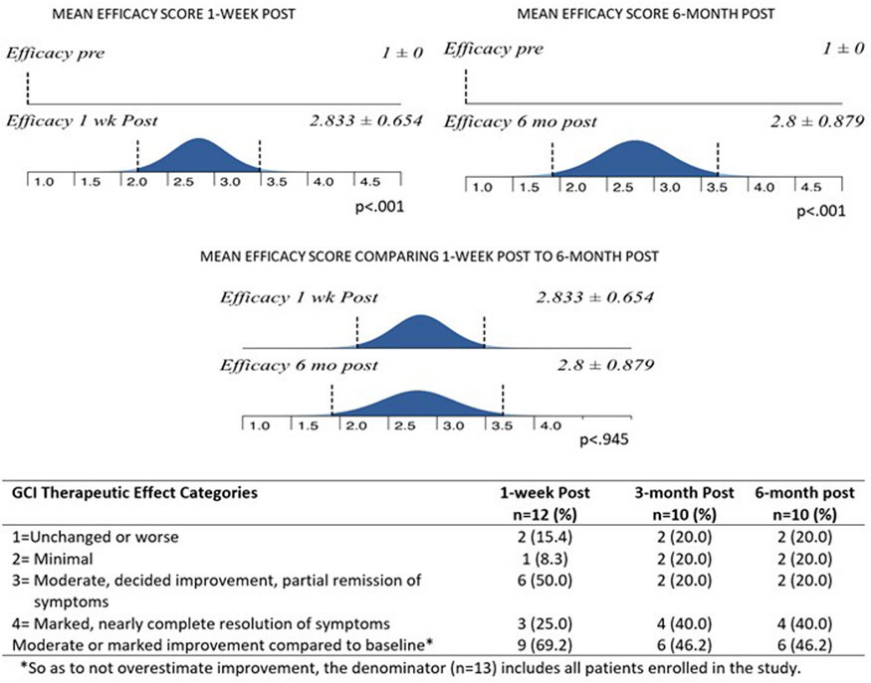
Mean therapeutic effect scores (efficacy) comparing baseline to after treatment.

Compared with baseline, changes in global improvement scores at 1 week and 6 months were statistically significant and depicted in Figure 4. The therapeutic effect at baseline was set at 0 = no change and compared with post-treatment in Figure 5. Even at 99% confidence, the SD never overlaps a CGI score that is associated with absence of improvement. Although there was significant correlation between lesser severity of illness and greater improvement, even the group of most severely ill women improved (*p* = 0.005).

### Short Form McGill Pain Questionnaire

**FIG. 6.**
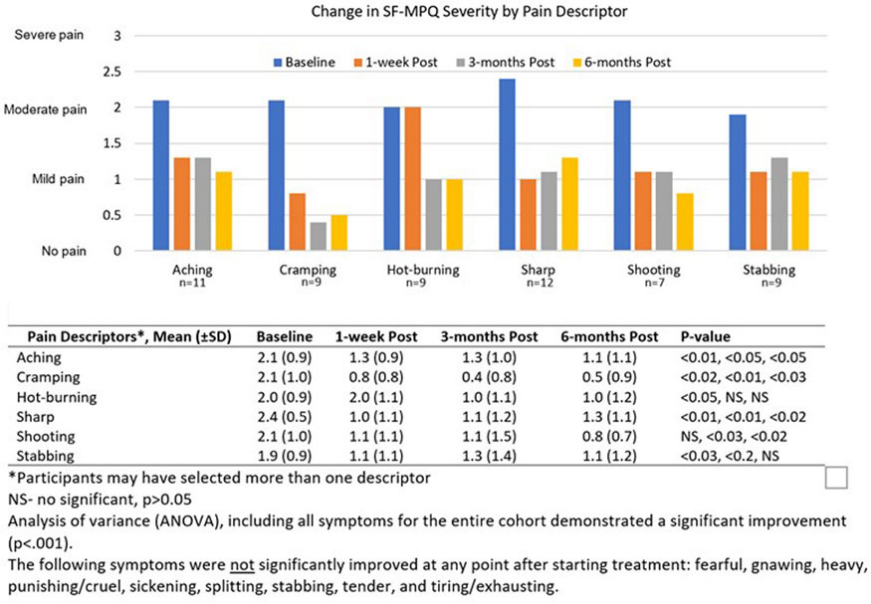
Change in SF-MPQ Mean Score for Each Pain Descriptor Across Time. SF-MPQ, Short Form-McGill Pain Questionnaire.

The prevalence of symptoms reported by the cohort at baseline is shown in Figure 6. At baseline, the mean SF-MPQ score was 19.7 (SD ±5.9). Compared with baseline, the mean SF-MPQ score decreased to 10.0 (SD ±7.5, *p* = 0.004, *d* = 1.6) at 1 week after treatment, to 9.7 (SD ±7.9, *p* = 0.005, *d* = 1.7) at 3 months, and 8.2 (SD ±8.1, *p* = 0.002, *d* = 1.9) at 6 months.

Among those subjects with CGI of greater than moderate pretreatment illness, there was no significant improvement noted in the following pain descriptors: gnawing (*n* = 5), fearful (*n* = 9), heavy (*n* = 4), hot-burning (*n* = 9), punishing-cruel (*n* = 3), sickening (*n* = 6), splitting (*n* = 4), tender (*n* = 12), or tiring (*n* = 5). However, compared with baseline, the same group of patients reported significant improvement aching (*n* = 11), cramping (*n* = 9), and sharp (*n* = 12), at all time points (Fig. 6). Hot-burning pain (*n* = 9) improved 1 week post-treatment; however, this effect was not sustained at 3 or 6 months (Fig. 6). Shooting (*n* = 7) pain did not significantly improve at 1 week, however, there was significant improvement at 3 and 6 months (Fig. 6).

## Discussion

In this pilot cohort, patients undergoing transvaginal PBM for pelvic pain demonstrated significant pain improvement as reported by both patients and clinicians. Even after accounting for three study participants lost to long-term follow-up, 60% of study participants were described as improved, and of those who improved, 75% were “much” or “very much” improved. On average, patients described 50% reduction in overall pain severity and this effect was sustained over 6 months.

The most common pain descriptors used by patients included sharp, tender, aching, stabbing, throbbing, cramping, and hot-burning. The biggest improvement in aching, cramping, hot-burning, shooting, and stabbing pain was noted as early as 1 week after treatment. At 3 months, participants reported significant improvement in aching, cramping, sharp, shooting, and stabbing pain. Except for stabbing and hot-burning pain, all other pain descriptors remained improved at 6 months. Mean severity levels indicate that at baseline, patients experience moderate levels of pain, however, after treatment, pain severity was reduced to mild for all descriptors. We did not find an effect of PBM on 8 of the 15 pain descriptors; this is likely due to the small sample size given that these descriptors were rarely used by our patients with CPP.

Adherence with therapy was high; 13 out of 14 patients invited to participate in the study completed all 9 treatments, 85% (*n* = 11) returned for follow-up 1 week later, and 54% and 62% returned for follow-up 3 and 6 months later. Throughout the study period, there were no serious adverse events reported by clinicians or patients.

These results are reassuring but must be interpreted with caution, as our study has all the limitations inherent to a pilot study: small sample size, lack of randomization, and lack of a control group. We also did not evaluate other outcomes besides pain, such as quality of life, sexual function, and psychological health. Nonetheless, it is notable that even within this small sample, the effect size for pain is rather large and sustained over 6 months suggesting that TV-PBMS may be an effective treatment for pelvic pain that deserves further study.

Since we did not exclude any pelvic pain diagnosis, it should be emphasized that improvement in pain was noted regardless of the primary cause of pain or additional pain comorbidities (e.g., IBS, IC/PBS, endometriosis). This finding is consistent with the mechanism action of PBMS and the ability of this TV-PMS to reach both the muscles and viscera of the pelvis.^37^ The results observed in this pilot study may be secondary to the ability of PBM to induce muscle relaxation, promote tissue regeneration and neovascularization, and reduce inflammation, all factors believed to contribute to CPP.

This comprehensive mechanism of action may have a therapeutic effect across a diverse group of pelvic pain patients with multiple pelvic pain-related diagnoses as long as they present with any of the following symptoms: generalized pelvic pain; pelvic pain with sitting, standing, or exercise; dyspareunia or vulvar pain; pain with bowel movements or urination; and other symptoms of pelvic dysfunction such as urgency or frequency.

In this study, we also found that TV-PBM was easily applied in office-based settings with a low risk for adverse events. If the therapeutic effect is confirmed in larger controlled trials, then TV-PBM has the potential to be widely deployed in clinical practice and improve access to pain therapy, even in areas where patients do not have access to a pain specialist.

## Conclusion

Transvaginal PBM administered with the SoLá Pelvic Therapy System significantly reduced pelvic pain in this pilot study population. This effect was maintained at 6 months following treatment. Future controlled studies will need to determine if TV-PBM is a safe and effective treatment for women with CPP conditions. Much research still needs to be done, and we hope that the data presented in this study provide the baseline information needed to conduct larger controlled trials.

## Ethics Approval

This study was approved by the New England Institutional Review Board on August 19, 2009; IRB approval number 09-152.

## Abbreviations Used

ACOG: American College of Obstetricians and Gynecologists
ATP: adenosine triphosphate
CGI: Clinical Global Impression
COX: cytochrome c oxidase
CPP: chronic pelvic pain
FDA: Food and Drug Administration
IBS: irritable bowel syndrome
IC/BPS: interstitial cystitis/bladder pain syndrome
LLLT: low-level laser therapy
NIR: near-infrared
PBM: photobiomodulation
PT: physical therapy
SD: standard deviation
SF-MPQ: Short Form-McGill Pain Questionnaire
TV-PBM: transvaginal PBM

## References

[1] Mathias SD, Kuppermann M, Liberman RF, Lipschutz RC, Steege JF. Chronic pelvic pain: Prevalence, health-related quality of life, and economic correlates. Obstet Gynecol 1996;87:321–327. 8598948 10.1016/0029-7844(95)00458-0

[2] Latthe P, Latthe M, Say L, Gulmezoglu M, Khan KS. WHO systematic review of prevalence of chronic pelvic pain: A neglected reproductive health morbidity. BMC Public Health 2006;6:177. 16824213 10.1186/1471-2458-6-177 PMC1550236

[3] American College of Obstetricians, Gynecologists' Committee on Practice Bulletins—Gynecology. Chronic Pelvic Pain: ACOG Practice Bulletin, Number 218. Obstet Gynecol 2020;135:e98–e109. 32080051 10.1097/AOG.0000000000003716

[4] Pain IAftSo. Classification of Chronic Pain, Second Edition (Revised). Available at: https://www.iasp-pain.org/PublicationsNews/Content.aspx?ItemNumber=1673&navltemNumber=677. Published 2020. Accessed April 7, 2021.

[5] Allaire C, Williams C, Bodmer-Roy S, et al. Chronic pelvic pain in an interdisciplinary setting: 1-year prospective cohort. Am J Obstet Gynecol 2018;218:114.e1–114.e12. 29031895 10.1016/j.ajog.2017.10.002

[6] Lamvu G, Williams R, Zolnoun D, et al. Long-term outcomes after surgical and nonsurgical management of chronic pelvic pain: One year after evaluation in a pelvic pain specialty clinic. Am J Obstet Gynecol 2006;195:591–598; discussion 598–600. 16729951 10.1016/j.ajog.2006.03.081

[7] Lamvu G, Carrillo J, Ouyang C, Rapkin A. Chronic pelvic pain in women: A review. JAMA 2021;325:2381–2391. 34128995 10.1001/jama.2021.2631

[8] Veasley C, Clare D, Clauw DJ, et al. Impact of Chronic Overlapping Pain Conditions on Public Health and the Urgent Need for Safe and Effective Treatment- 2015 Analysis and Policy Recommendations. Chronic Pain Research Alliance, 2020. Available at: http://www.chronicpainresearch.org/public/CPRA_WhitePaper_2015-FINAL-Digital.pdf. Published 2015. Accessed November 17, 2021.

[9] Zondervan KT, Yudkin PL, Vessey MP, et al. Chronic pelvic pain in the community—Symptoms, investigations, and diagnoses. Am J Obstet Gynecol 2001;184:1149–1155. 11349181 10.1067/mob.2001.112904

[10] Fitzgerald CM, Neville CE, Mallinson T, Badillo SA, Hynes CK, Tu FF. Pelvic floor muscle examination in female chronic pelvic pain. J Reprod Med 2011;56:117–122. 21542528

[11] Mieritz RM, Thorhauge K, Forman A, Mieritz HB, Hartvigsen J, Christensen HW. Musculoskeletal dysfunctions in patients with chronic pelvic pain: A preliminary descriptive survey. J Manipulative Physiol Ther 2016;39:616–622. 27776745 10.1016/j.jmpt.2016.09.003

[12] Sedighimehr N, Manshadi FD, Shokouhi N, Baghban AA. Pelvic musculoskeletal dysfunctions in women with and without chronic pelvic pain. J Bodyw Mov Ther 2018;22:92–96. 29332764 10.1016/j.jbmt.2017.05.001

[13] Rodriguez MA, Afari N, Buchwald DS; National Institute of Diabetes and Digestive and Kidney Diseases Working Group on Urological Chronic Pelvic Pain. Evidence for overlap between urological and nonurological unexplained clinical conditions. J Urol 2009;182:2123–2131. 19758633 10.1016/j.juro.2009.07.036 PMC2957306

[14] Tirlapur SA, Kuhrt K, Chaliha C, Ball E, Meads C, Khan KS. The ‘evil twin syndrome’ in chronic pelvic pain: A systematic review of prevalence studies of bladder pain syndrome and endometriosis. Int J Surg 2013;11:233–237. 23419614 10.1016/j.ijsu.2013.02.003

[15] Zondervan KT, Yudkin PL, Vessey MP, et al. The community prevalence of chronic pelvic pain in women and associated illness behaviour. Br J Gen Pract 2001;51:541–547. 11462313 PMC1314045

[16] Berlanda N, Vercellini P, Fedele L. The outcomes of repeat surgery for recurrent symptomatic endometriosis. Curr Opin Obstet Gynecol 2010;22:320–325. 20543689 10.1097/GCO.0b013e32833bea15

[17] Flyckt R, Kim S, Falcone T. Surgical management of endometriosis in patients with chronic pelvic pain. Semin Reprod Med 2017;35:54–64. 28049215 10.1055/s-0036-1597306

[18] Hartmann KE, Ma C, Lamvu GM, Langenberg PW, Steege JF, Kjerulff KH. Quality of life and sexual function after hysterectomy in women with preoperative pain and depression. Obstet Gynecol 2004;104:701–709. 15458889 10.1097/01.AOG.0000140684.37428.48

[19] Fuentes-Marquez P, Cabrera-Martos I, Valenza MC. Physiotherapy interventions for patients with chronic pelvic pain: A systematic review of the literature. Physiother Theory Pract 2019;35:1131–1138. 29757068 10.1080/09593985.2018.1472687

[20] Yunker A, Sathe NA, Reynolds WS, Likis FE, Andrews J. Systematic review of therapies for noncyclic chronic pelvic pain in women. Obstet Gynecol Surv 2012;67:417–425. 22926248 10.1097/OGX.0b013e31825cecb3

[21] Cotler HB, Chow RT, Hamblin MR, Carroll J. The use of low level laser therapy (LLLT) for musculoskeletal pain. MOJ Orthop Rheumatol 2015;2:00068. 26858986 10.15406/mojor.2015.02.00068 PMC4743666

[22] Clijsen R, Brunner A, Barbero M, Clarys P, Taeymans J. Effects of low-level laser therapy on pain in patients with musculoskeletal disorders: A systematic review and meta-analysis. Eur J Phys Rehabil Med 2017;53:603–610. 28145397 10.23736/S1973-9087.17.04432-X

[23] Glazov G, Yelland M, Emery J. Low-level laser therapy for chronic non-specific low back pain: A meta-analysis of randomised controlled trials. Acupunct Med 2016;34:328–341. 27207675 10.1136/acupmed-2015-011036 PMC5099186

[24] Yeh SW, Hong CH, Shih MC, Tam KW, Huang YH, Kuan YC. Low-level laser therapy for fibromyalgia: A systematic review and meta-analysis. Pain Physician 2019;22:241–254. 31151332

[25] Young S, Bolton P, Dyson M, Harvey W, Diamantopoulos C. Macrophage responsiveness to light therapy. Lasers Surg Med 1989;9:497–505. 2811573 10.1002/lsm.1900090513

[26] Anders JR, T, Moges H, Ilev I, Waynant R, Longo L. Light Interaction With Human Central Nervous System Progenitor Cells. NAALT conference proceedings; Tucson, Arizona, 2007.

[27] Gigo-Benato D, Russo TL, Tanaka EH, Assis L, Salvini TF, Parizotto NA. Effects of 660 and 780 nm low-level laser therapy on neuromuscular recovery after crush injury in rat sciatic nerve. Lasers Surg Med 2010;42:673–682. 20976807 10.1002/lsm.20978

[28] Rochkind S, Leider-Trejo L, Nissan M, Shamir MH, Kharenko O, Alon M. Efficacy of 780-nm laser phototherapy on peripheral nerve regeneration after neurotube reconstruction procedure (double-blind randomized study). Photomed Laser Surg 2007;25:137–143. 17603852 10.1089/pho.2007.2076

[29] Rochkind S, Drory V, Alon M, Nissan M, Ouaknine GE. Laser phototherapy (780 nm), a new modality in treatment of long-term incomplete peripheral nerve injury: A randomized double-blind placebo-controlled study. Photomed Laser Surg 2007;25:436–442. 17975958 10.1089/pho.2007.2093

[30] Litecure L, ed. Litecure LCT-1000 User Manual. Newark, DE: Litecure, LLC, 19702.

[31] Pendergrass PB, Belovicz MW, Reeves CA. Surface area of the human vagina as measured from vinyl polysiloxane casts. Gynecol Obstet Invest 2003;55:110–113. 12771458 10.1159/000070184

[32] Tan JS, Lukacz ES, Menefee SA, Luber KM, Albo ME, Nager CW. Determinants of vaginal length. Am J Obstet Gynecol 2006;195:1846–1850. 17014819 10.1016/j.ajog.2006.06.063

[33] Melzack R. The short-form McGill Pain Questionnaire. Pain 1987;30:191–197. 3670870 10.1016/0304-3959(87)91074-8

[34] Busner J, Targum SD. The Clinical Global Improvement Scale: Applying a research tool in clinical practice. Psychiatry 2007;4:28–37. PMC2880930 20526405

[35] Todd KH. Clinical versus statistical significance in the assessment of pain relief. Ann Emerg Med 1996;27:439–441. 8604855 10.1016/s0196-0644(96)70226-3

[36] Younger J, McCue R, Mackey S. Pain outcomes: A brief review of instruments and techniques. Curr Pain Headache Rep 2009;13:39–43. 19126370 10.1007/s11916-009-0009-x PMC2891384

[37] Zipper R, Pryor B. Evaluation of a novel deep tissue transvaginal near-infrared laser and applicator in an ovine model. Lasers Med Sci 2021. https://link.springer.com/article/10.1007%2Fs10103-021-03315-z 10.1007/s10103-021-03315-z PMC8803674 33855615

